# Etiologic characteristics of avian influenza H11 viruses isolated from the live poultry market in southeast coastal region in China

**DOI:** 10.3389/fmicb.2022.1002670

**Published:** 2022-10-21

**Authors:** Lina Jiang, Jiaming Li, Huan Cui, Cheng Zhang, Yifei Jin, Yingying Fu, Ningning Ma, Fei Tang, Yidun Zhang, Jing Zheng, Li Li, Bing Lu, Zehui Chen, Zhendong Guo, Zhongyi Wang

**Affiliations:** ^1^Xiamen Center for Disease Control and Prevention, Xiamen, China; ^2^Beijing Institute of Biotechnology, Beijing, China; ^3^Changchun Veterinary Research Institute, Chinese Academy of Agriculture Sciences, Changchun, China; ^4^Beijing Institute of Health Care, Beijing, China

**Keywords:** H11N3 influenza virus, rare subtypes, live poultry market, mammals, transmission

## Abstract

Since it was first identified in 1956, the H11 subvariant influenza virus has been reported worldwide. However, due to the low pathogenicity of the H11 subvariant and the absence of its widespread transmission among humans, there are only a few reports on the etiology of the H11 subvariant influenza virus. Therefore, in the present study, we isolated a strain of the H11N3 avian influenza virus (AIV) from poultry feces from the live poultry market in the southeast coastal region of China. Considering that the H11 subvariant is known to cause infections in humans and to enrich the knowledge of the H11 subvariant of the avian influenza virus, the genetics, pathogenicity, and transmissibility of the isolate were studied. The phylogenetic analysis indicated that the H11N3 isolate was of Eurasian origin and carried genes closely related to duck H7N2 and H4N6. The receptor binding analysis revealed that the H11N3 isolate only acquired a binding affinity for avian-derived receptors. In the respiratory system of mice, the isolate could directly cause infection without adaptation. In addition, the results from transmission experiments and antibody detection in guinea pigs demonstrated that H11N3 influenza viruses can efficiently transmit through the respiratory tract in mammalian models. Direct infection of the H11N3 influenza virus without adaptation in the mouse models and aerosol transmission between guinea pig models confirms its pandemic potential in mammals, underscoring the importance of monitoring rare influenza virus subtypes in future studies.

## Introduction

The avian influenza virus (AIV) is a member of the Orthomyxoviridae family isolated from and adapted to an avian host. Influenza A virus has 18 HA subtypes and 11 NA subtypes according to its hemagglutinin (HA) and neuraminidase (NA) differences (Spackman, 2014). Waterfowl and birds are the natural reservoirs of avian influenza viruses (Wang et al., 2018). In 1956, the H11 subtype of avian influenza virus was first isolated from domestic ducks in England (Webster et al., 1992). Following that, isolation of this subtype of the virus has been reported in several countries around the world. The H11 subtype of AIV is a low pathogenic avian influenza virus (LPAIV) covering nine different NA subtypes. Pawar et al. and Koratkar et al. isolated the H11N1 avian influenza virus from the Eurasian spoonbill in India (Koratkar et al., 2014) and from *Anas acuta* in Japan (Pawar et al., 2010), respectively. Yuee Zhang et al. isolated an H11N2 subtype influenza virus from samples collected from the live poultry market in east China and performed whole-genome sequencing (Zhang et al., 2012). The H11 subtype is most often co-infected with other subtypes of the influenza virus and has not been found to show significant clinical symptoms in infected ducks but has been reported to infect mammals—even humans (Gill et al., 2006). Data from GenBank showed that A/swine/KU/2/2001 (H11N6) was isolated from domestic pigs in Korea, proving that the subtype virus has the potential to infect mammals (Tuong et al., 2020). Antibodies to the H11 subtype virus have been repeatedly detected in the serum of poultry (Kayali et al., 2011; Luo et al., 2021). The continuous spread of this virus could pose a potential threat.

The live poultry market (LPM) is a gathering and distributing place for poultry, which is characterized by a large number of poultry species from complex sources, involving long-distance transportation and frequent contact between humans and poultry, which plays a key role in the spread and evolution of AIV (Wei et al., 2019). In the present study, a strain of the H11N3 subtype of AIV was isolated from ducks in a live poultry market in Fujian Province, China. Considering that the H11 subvariants were known to cause human infections, we investigated the viruses *in vitro* and *in vivo* (Gill et al., 2006; Kayali et al., 2011). Through whole-genome sequencing, genetic evolution analysis, and mouse infection and transmission experiments of the strain, the genetic evolution status of the virus was clarified, and the infectious risk of the virus to mammals was assessed. This study provides a reference for the comprehensive prevention and control of the H11 subtype.

## Materials and methods

### Ethical statement

The study was conducted strictly according to the Guidelines for the Care and Use of Laboratory Animals provided by the Ministry of Science and Technology of China. The study protocol was reviewed and approved by the Animal Experiment Ethics Committee of Changchun Veterinary Research Institute, Chinese Academy of Agricultural Sciences. The H5N1 virus experiments were conducted in an animal biosafety level 3 (ABSL-3) laboratory.

### Virus isolation

We collected environmental samples on 14 January 2020 at Xindian Farmers Market, Xiang'an district, Xiamen city. After preliminary screening of the samples by real-time PCR, the influenza A virus was detected in a poultry fecal sample, which was temporarily named EV01.

After shaking and centrifugation of the virus-detected sample, the supernatant was harvested and inoculated with 10-day-old SPF chicken embryos, and allantoic fluid was collected after culturing at 37°C for 48 h. The isolated virus was serially passaged three times in 10-day-old SPF chicken embryos, and the allantoic fluid of the chicken embryos was harvested and stored at −80°C.

### Genetic and phylogenetic analyses

TRIzol reagent (TaKaRa) was used to extract total RNA from the allantoic fluid, and a PrimeScript^TM^ RT Reagent Kit (TaKaRa) was used to prepare cDNA using an influenza universal oligonucleotide primer Uni12 (Supplementary Table 1). The obtained cDNA was sent to Shanghai Sangon Bioengineering Co., Ltd. for specific primer PCR of the viral genome, and the resultant PCR product was sequenced. Based on the sequences downloaded from GenBank, we compared the reference sequences of HA and NA genes with the sequences of the strains obtained in this study using the ClustalW method. The GTRGAMMA nucleotide substitution model in PhyML 3.1 software was used, and bootstrap replicates were run 1,000 times to evaluate the maximum likelihood (ML) phylogenies of codons of the two gene sequences. Phylogenetic trees were visualized using FigTree v1.4.4. The nucleotide sequences obtained in this study have been submitted to the GenBank database (accession numbers ON968457–ON968464).

### Identification of receptor binding specificity

The receptor binding affinities of the EV01 virus were determined by performing HA assays using 1% desialylated chicken red blood cells (cRBCs; Zhang X. et al., 2021). Specifically, to enzymatically remove sialic acid residues from cRBCs, the cells were treated with 50 mU of vibrio cholerae neuraminidase (VCNA) for 1 h, and then, the cells were resialylated using α-2,6-(N)-sialyltransferase or α-2,3-(N)-sialyltransferase (Sigma-Aldrich) at 37°C for 3 h. In addition, an HA assay was performed on VCNA-treated cRBCs, resialylated cRBCs, and normal cRBCs using 4 different erythrocytes to analyze the receptor binding specificity of the virus. A/chicken/Hebei/HB777/2006(HB777[H5N1]) virus isolates from poultry and A/California/04/2009 (CA04[H1N1]) virus isolates from humans were used as controls for preferential binding to avian-type SA α-2,3-Gal and human-type SA α-2,6-Gal, respectively.

### Growth dynamics in cells

A549 and MDCK cells were used to compare EV01 growth kinetics. A multiplicity of infection (MOI) of 0.001 was used to inoculate the virus into two cell monolayers. Infected cells were harvested at 12, 24, 36, 48, and 60 h post-inoculation (h.p.i.) and stored at −80°C, and then, virus titers were determined by median egg infective dose (EID_50_) assays.

### Experimental infection of mice

Mouse infection experiments were conducted to evaluate the pathogenicity of the virus in a mammalian host. For the experiment, 6-week-old female BALB/c mice were purchased from Beijing Vital River Laboratory Animal Technology Co., Ltd. Mice infection experiments were divided into two parts. The first part is weight monitoring, where 10 mice were equally divided into two groups: the infection group and the negative control group. The two groups of mice were anesthetized with isoflurane and were nasally inoculated with 50 μL of PBS buffer and 10^6^ EID_50_ of the virus. A 14-day continuous weight monitoring program was instituted for all mice of the two groups. The second part of the mice infection experiment was the *in vivo* monitoring of virus replication. We randomly divided 15 mice into two groups, namely, the infection group and the negative control group, the same as in the first part. Virus infection was conducted based on the protocol described previously (Zhang C. et al., 2021). The mice in the infected group were euthanized at 1, 3, 5, and 7 day(s) post-inoculation (d.p.i.) to measure viral replication. A total of 10 tissue samples, namely, the heart, the liver, the spleen, the lung, the kidney, the brain, the trachea, the pancreas, the intestine, and the turbinate, were collected, and the samples were homogenized in 1 ml of PBS with 1% penicillin–streptomycin. Viral multiplication was determined in each tissue by EID_50_ assays. Then, we evaluated the clinical symptoms associated with viral infection in mouse models. For histopathological examination, a part of the lung tissue was fixed with 4% paraformaldehyde at 3 d.p.i.

### Evaluation of transmissibility among guinea pigs

For the study of EV01 virus transmission in mammals, nine female guinea pigs of SPF Hartley strain weighing 300–350 g were purchased from Beijing Vital River Laboratory Animal Technology Co., Ltd. Of the nine guinea pigs, three of them were inoculated intranasally with 10^6^ EID_50_ of EV01 virus at a volume of 200 μl (100 μl per nostril), which were considered the infection group. All the guinea pigs were transferred to a “tailor-made transmissibility evaluation cage” at 1 d.p.i. To study the direct contact transmission capacity of the virus, three unvaccinated guinea pigs were placed in the same isolator as the three guinea pigs of the infected group. Meanwhile, to monitor the spread of aerosols, three additional unvaccinated guinea pigs were kept in an adjacent isolator (5 cm apart). At 1, 3, 5, and 7 d.p.i., nasal wash samples were harvested using 1 ml PBS with 1% penicillin–streptomycin and titrated in SPF embryonic eggs for EID_50_ testing.

### Antibody detection

Serum was collected from all guinea pigs in as mentioned previously (Section Evaluation of transmissibility among guinea pigs) at 21 d.p.i., and the HI test was conducted according to the protocol described in the OIE Manual of Diagnostic Tests and Vaccines for Terrestrial Animals. A/California/04/2009 (CA04[H1N1]) virus isolates from humans were used as the control group in this study.

### Statistical analysis

GraphPad Prism was used to determine statistically significant differences using a one-way analysis of variance (ANOVA). Analyses were performed in triplicate and are representative of three separate experiments. The standard deviation is represented by error bars.

## Results

### Phylogenetic analysis

**Figure 1:**
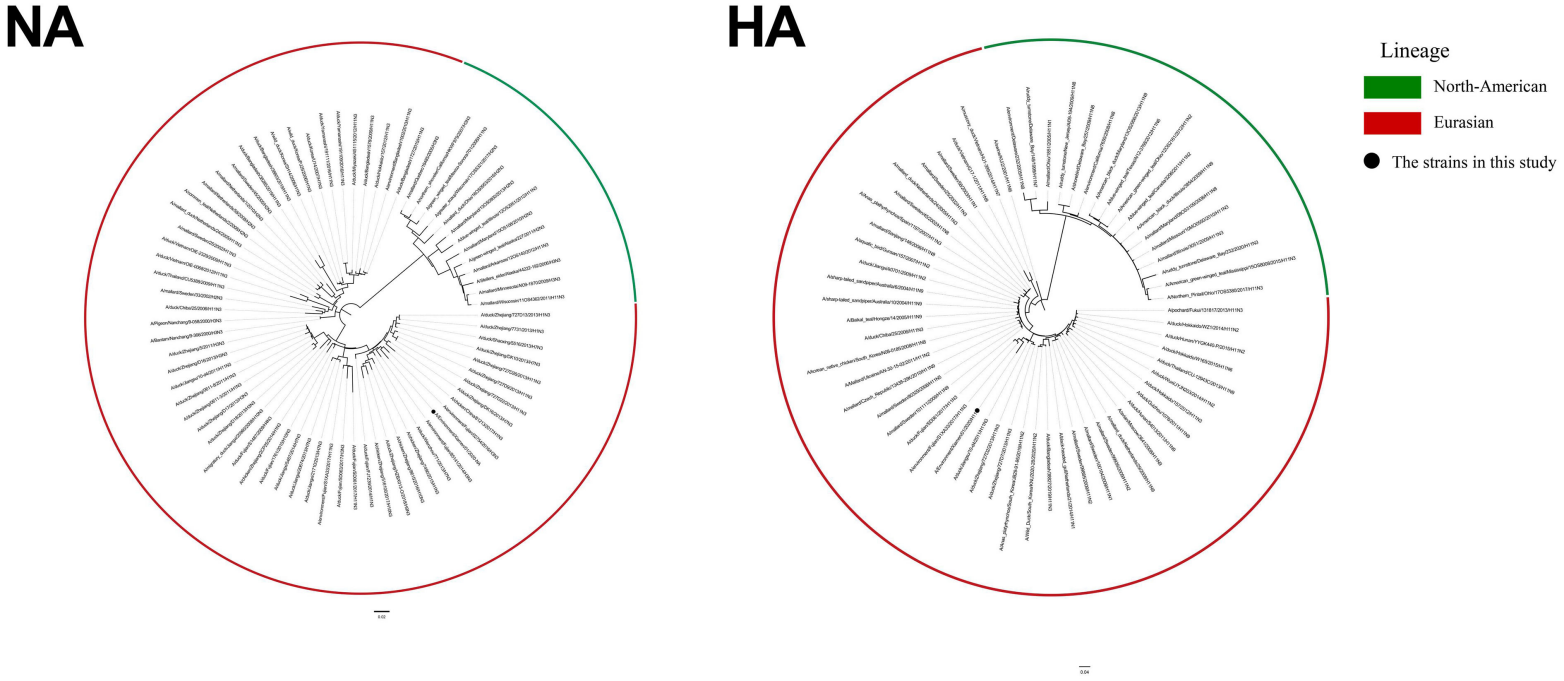
ML tree of HA and NA of H11N3 virus. The GTRGAMMA nucleotide substitution model in PhyML 3.1 software was used, and bootstrap replicates were run 1,000 times to evaluate the maximum likelihood (ML) phylogenies of codon comparison between the two gene sequences. Phylogenetic trees were visualized using FigTree v1.4.4. Black dots indicate the isolate EV01 in this study.

We performed a phylogenetic analysis of the genomes of the H11N3 virus using phylogenetic trees generated by PhyML 3.1 and FigTree v1.4.4 to acquire phylogenetic information. The phylogenetic analysis indicated that the H11N3 isolate was of Eurasian origin. EV01 virus isolates of HA and NA clustered into clade A/environment/Fujian/S1XA33/2017/H11N3 and clade A/environment/Fujian/02754/2016/H3N3, respectively (Figure 1). PB1, PB2, PA, M, NS, and NP clustered into clade A/chicken/Zhejiang/51048/2015/H1N9, A/duck/Fujian/SE0195/2018/H7N2, A/duck/Fujian/SD061/ 2017/H11N3, A/duck/Fujian/FZHX0004-C/2017/mixed, A/duck/Mongolia/926/2019/H5N3, and A/duck/Zhejiang/ 422/2013/H4N6 (Supplementary Figure 1).

### Test for receptor-binding property

**Figure 2:**
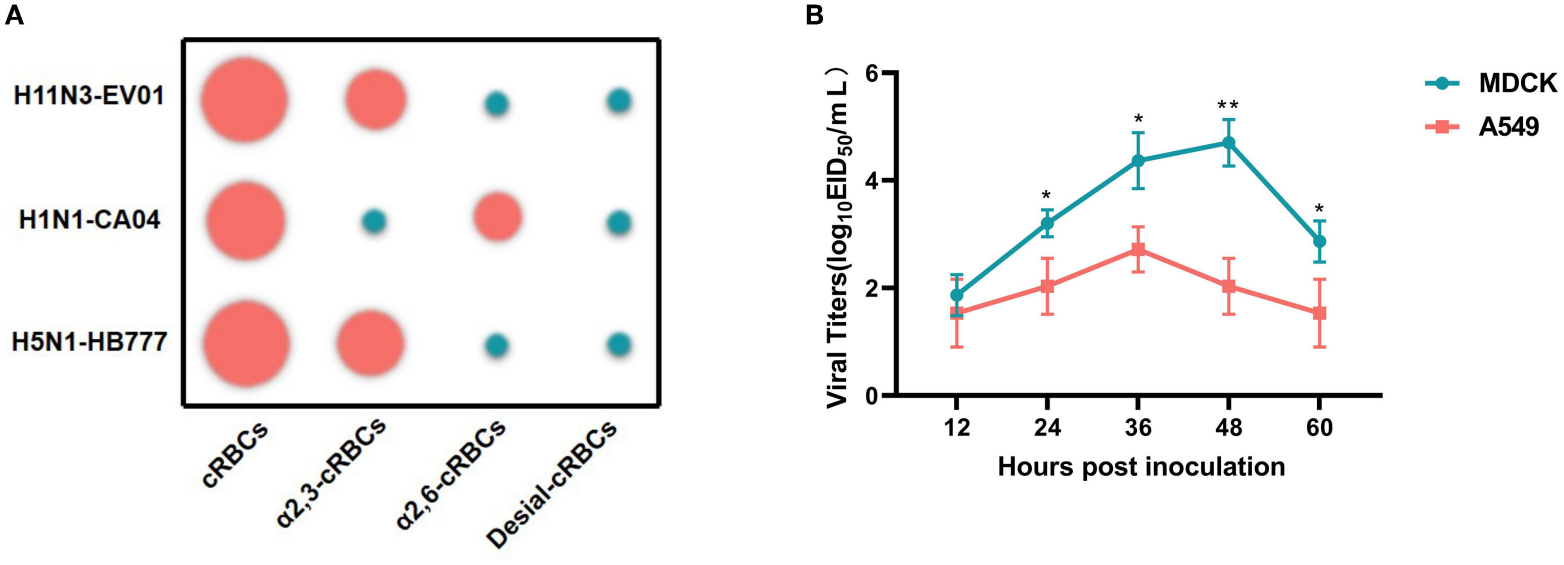
Receptor binding property of three IAV strains and replication kinetics of EV01 virus in MDCK and A549 cells. **(A)** Receptor binding property of three IAV strains. Green indicates a negative result, and red indicates a certain binding ability; the larger the circle, the stronger the binding ability. **(B)** Replication kinetics of EV01 virus in MDCK and A549 cells. The green broken line indicates the proliferation curve of EV01 in MDCK cells, and the red broken line indicates the proliferation curve of EV01 in A549 cells. Y-axis indicates viral titers in cells that have been infected with 0.001 MOI of the virus. Each data point on the polyline indicates the means and standard deviation of three independent experiments. Statistical significance between the two groups was calculated by unpaired Student's *t*-test. Values are expressed as the mean ± standard error of the mean (SEM). *P < 0.05, **P < 0.01.

The specificity of receptor binding is an important factor for IAV to cross species barriers. For avian influenza to cross species barriers, a-2,3-linked receptors must switch to a-2,6-linked receptors. HA assays were used to determine the receptor specificity of the EV01 virus, where the poultry-isolated A/chicken/Hebei/HB777/2006(HB777[H5N1]) and human-isolated A/California/04/2009 (CA04[H1N1]) viruses were used as controls for preferential binding. EV01 virus binds more readily to SA a-2,3 receptors according to the receptor binding property studies (Figure 2A, Supplementary Table 2).

### Analysis of replication kinetics

A549 and MDCK cells were used to study the replication kinetics of EV01. The result indicates that the EV01 virus showed a certain proliferation ability in both cells. However, from 24 h.p.i., the proliferation ability of the virus in MDCK cells was significantly higher than that in A549 cells (Figure 2B).

### Pathogenicity of EV01 virus in mice

**Figure 3:**
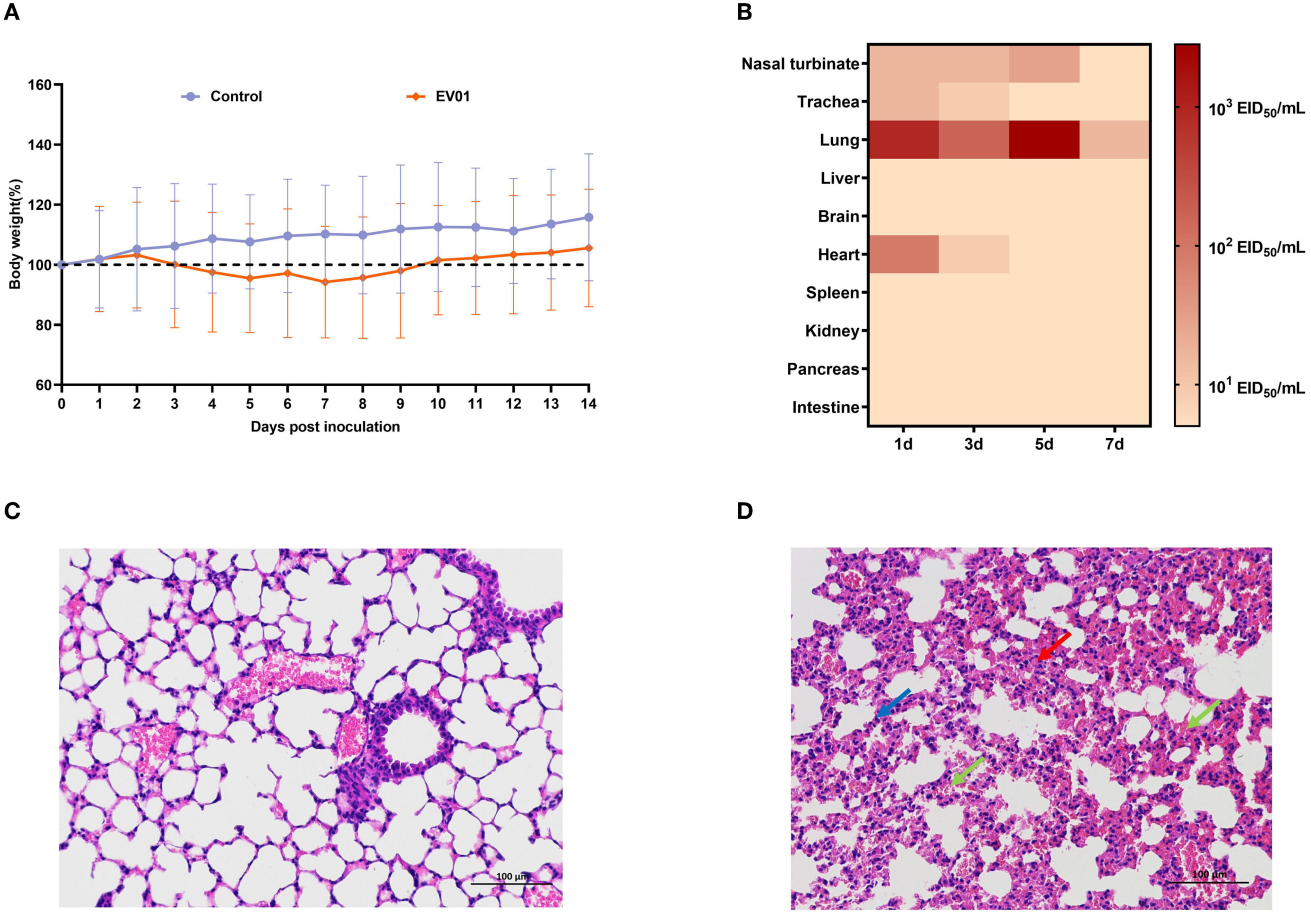
Pathogenicity of EV01 virus in mice. **(A)** Body weights were monitored daily over 14 days after inoculation. **(B)** Virus titers in different tissues of mice at 1, 3, 5, and 7 days post-inoculation with EV01 virus; darker colors indicate higher virus titers. **(C,D)** Lung tissue sections of healthy mice and mice infected with the EV01 virus. The sections were stained with H&E. The histopathological analysis of pulmonary tissues was acquired at ×20 magnification; blue arrows indicate inflammatory cell infiltration, green arrows indicate pus cells, and red arrows indicate alveolar wall thickening.

No noticeable clinical symptoms were observed in infected mice, but the body weight of the mice decreased slightly from 3 d.p.i. and resumed slow growth to 7 d.p.i. All the infected mice survived 14 days (Figure 3A, Supplementary Table 3). Viruses were found in the turbinates, the trachea, the pulmonary tissues, and the heart but not in the liver, the brain, the spleen, the kidney, the pancreas, or the intestines. The highest viral titer was found in the pulmonary tissue at 5 d.p.i., with a value of 10^3.53^ EID_50_/ml. The viral titer was lower in the trachea, consistent with the receptor-binding property test results in Section Test for receptor-binding property (Figure 3B, Supplementary Table 4). The pathological damage of the mice inoculated with either EV01 or PBS virus was assessed by staining pulmonary tissues with H&E. The pulmonary tissues of the control group inoculated with PBS were normal (Figure 3C), and the pulmonary tissues from the mice inoculated with EV01 virus showed alveolar wall thickening and inflammatory cell infiltration (Figure 3D).

### Transmissibility of EV01 virus in guinea pigs

**Figure 4:**
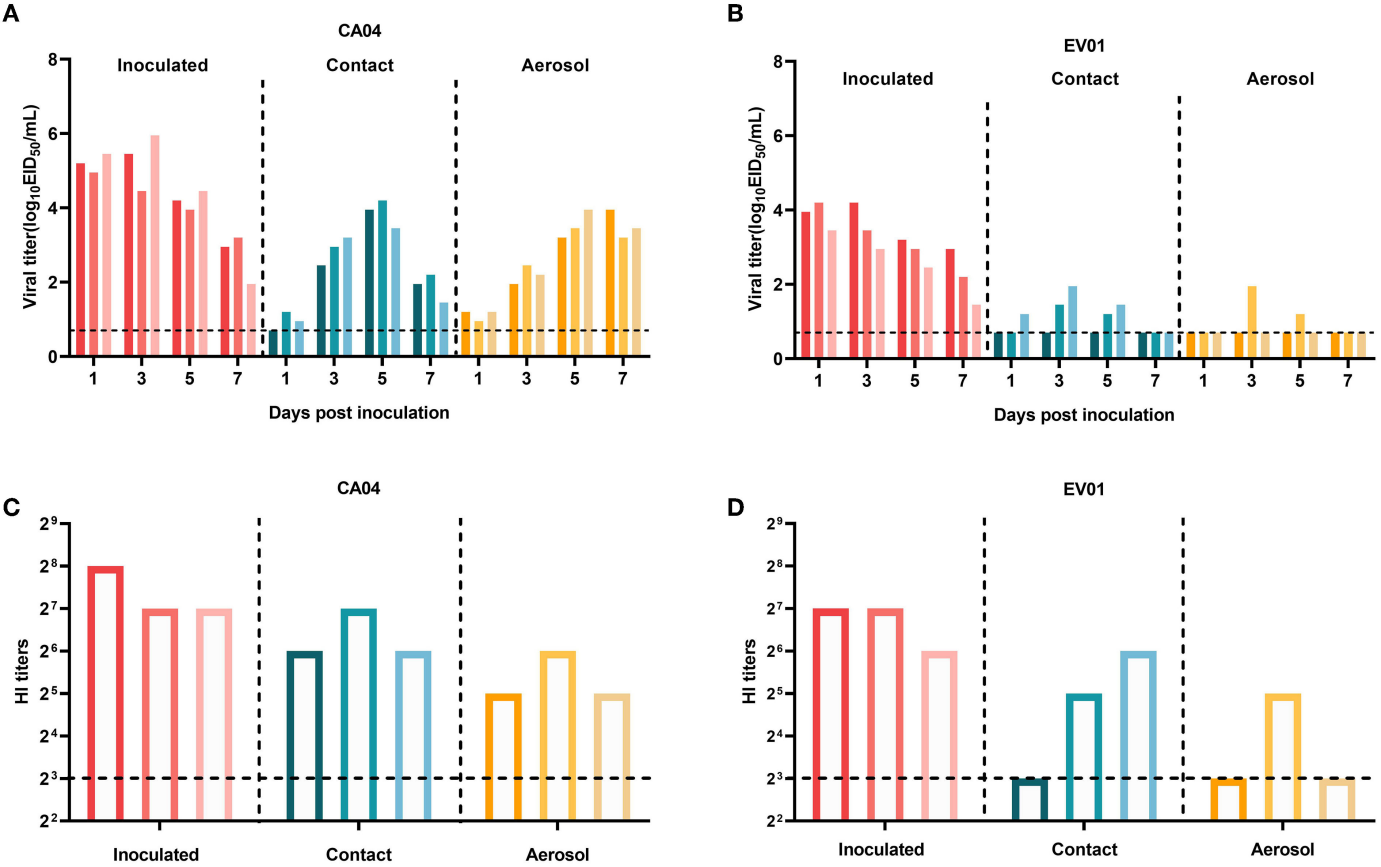
Evaluation of the transmissibility of the EV01 virus among guinea pigs. **(A)** Viral titers of the CA04 group. **(B)** Viral titers of the EV01 group. **(C)** Serum detection of each guinea pig in the CA04 group. **(D)** Serum detection of each guinea pig in the EV01 group. A total of three guinea pigs were inoculated with 200 μl of 10^6^ EID_50_ viruses (100 μl per nostril). After 24 h, the three naive guinea pigs were placed in the same cage as the infected pigs to see if direct contact transmission occurs, and three other naive guinea pigs were placed in an adjacent cage (with an interval of 5 cm) to observe respiratory droplet transmission. For the detection of virus shedding, nasal washes were collected every other day after inoculation for 1, 3, 5, and 7 days after inoculation. Serum was collected at 21 d.p.i., and the HI test was conducted according to the protocol described in the OIE Manual of Diagnostic Tests and Vaccines for Terrestrial Animals. Red, green, and yellow indicate the inoculated group, the contact group, and the aerosol group, respectively. The different shades of color bars in each group indicate individual animals, and the dashed line indicates the lower limit of detection.

The CA04 virus readily infected guinea pigs, as shown in Figure 4A. At 1 d.p.i. with CA04, guinea pigs had a nasal wash titer of 4.95–5.45 lgEID_50_/ml. In the inoculated guinea pigs, the virus titer peaked at 3 d.p.i. with a titer of 5.95 lgEID_50_/ml. Detectable nasal wash virus titers were relatively low in contract and exposed animals, peaking at 4.20 and 3.95 lgEID_50_/ml, respectively. The virus was detected in nasal washes of all groups, indicating efficient horizontal transmission, which is consistent with previous research (Itoh et al., 2009). The seroconversion of all guinea pigs was consistent with virus transmission (Figure 4C). These findings are consistent with those of previous studies, demonstrating the validity of the research method. Figure 4B shows that the EV01 virus can also infect guinea pigs. EV01-inoculated guinea pigs showed a nasal wash titer from 3.45 to 4.20 lgEID_50_/ml at 1 d.p.i. In the inoculated guinea pigs, the virus titer peaked at 3 d.p.i., with a titer of 4.20 lgEID_50_/ml. Detectable nasal wash virus titers were relatively low in contract and exposed animals, and the peak value of each group was 1.95 lgEID_50_/mL. In total, three of the inoculated guinea pigs were positive for the virus, due to direct contact with two pigs, and for virus shedding in one guinea pig. Seroconversion results are identical to virus-shedding test results in both groups (Figure 4D). The results showed that the EV01 virus can be aerosol-transmitted between guinea pigs. In summary, the EV01 virus was efficient in replicating in guinea pigs as well as in transmitting efficiently in contact and exposed guinea pigs.

## Discussion

Recently, some popular subtypes of the influenza virus (e.g., H9N2 and H5N6) have been confirmed to infect humans, and research on these influenza virus subtypes has also emerged endlessly (Peiris et al., 1999; Schwartz et al., 2019; Li et al., 2022). Researchers in many countries reported the H11 subtype of the avian influenza virus. However, in-depth research on the H11 subtype of the avian influenza virus is rarely reported. Therefore, in the present study, we isolated the avian influenza H11N3 virus from the poultry market environment in the southeast coastal region of China and systematically studied the evolutionary origin, pathogenicity, and transmission ability of the strain.

Phylogenetic analysis demonstrated that all eight genes of EV01 belong to the Eurasian lineage. The genes isolated in the current study have high homology with genes of some subtype strains found in Zhejiang, Mongolia, and other places. This study revealed that the H11N3 subtype of the avian influenza virus may undergo different types of recombination in different regions. This may be due to the migration of birds or the spread of the virus *via* the poultry supply chain.

The test for receptor-binding property demonstrated that EV01, like HB777[H5N1] (Zhao et al., 2013), only has α-2,3 receptor binding capacity, indicating a lower risk of cross-species infection. MDCK cells are commonly used to study influenza virus replication because they possess both avian- and human-like receptors. In previous studies, A549 cells also have been proven as a useful cell line to study the infectivity and replication of influenza viruses (Zhu et al., 2015; Shi et al., 2016). As expected, our study found that the EV01 virus showed better proliferative ability in MDCK cells than in A549 cells. These results are consistent with the receptor-binding property.

Moreover, *in vivo* study results are often inconsistent with *in vitro* study results, which necessitates the development of an ideal mammalian cell model to assess infectivity and replication. Mice and guinea pigs are considered ideal models for AIV adaptation and transmission (Schulman, 1968; Samira et al., 2009; Rodriguez et al., 2017). In our study, EV01 is pathogenic to mice and persists in their lungs for a long time. The results of the guinea pig transmission study and the serological study showed that EV01 has a certain risk of host-to-host transmission, with a direct contact transmission efficiency of 2/3 and aerosol transmission efficiency of 1/3.

Based on the aforementioned results, we speculate that H11N3 can cause disease in mammals after adaptation. Strengthening surveillance to prevent cross-species infections and human pandemics and avoiding biosafety risks are recommended. Overall, populations of RNA viruses exhibit large genetic variability; even uncommon low pathogenic influenza viruses have strong epidemic potential in a population. Rare subtypes of the virus should be monitored to prevent them from quietly developing major variants and mammalian pathogenicity due to poor surveillance.

## Data availability statement

The datasets presented in this study can be found in online repositories. The names of the repository/repositories and accession number(s) can be found at: https://www.ncbi.nlm.nih.gov/genbank/, ON968457 – ON968464.

## Ethics statement

The animal study was reviewed and approved by Animal Experiment Ethics Committee of Changchun Veterinary Research Institute, Chinese Academy of Agricultural Sciences.

## Author contributions

ZW, ZG, and ZC contributed to conception and design of the study. LJ, HC, CZ, FT, YZ, JZ, and LL performed some of the experiments. NM performed the statistical analysis. JL wrote the first draft of the manuscript. YJ, YF, and BL wrote sections of the manuscript. All authors contributed to manuscript revision, read, and approved the submitted version.

## Funding

This study was financially supported by the Beijing Science and Technology New Star Program (Z211100002121064), the Fujian Province Health Science and Technology Project (2020CXB050), and the Key Specialty of Clinical Medicine of Xiamen, China (Xiamen Health Commission Science and Education Department [2021] No. 215).

## Conflict of interest

The authors declare that the research was conducted in the absence of any commercial or financial relationships that could be construed as a potential conflict of interest.

## Publisher's note

All claims expressed in this article are solely those of the authors and do not necessarily represent those of their affiliated organizations, or those of the publisher, the editors and the reviewers. Any product that may be evaluated in this article, or claim that may be made by its manufacturer, is not guaranteed or endorsed by the publisher.
