# Supplementary material for: Etiologic characteristics of avian influenza H11 viruses isolated from the live poultry market in southeast coastal region in China
Source: Front Microbiol. 2022 Oct 21;13:1002670. doi: 10.3389/fmicb.2022.1002670 (PMC9634483; doi:10.3389/fmicb.2022.1002670)
Supplement: Supplementary file 1 [file Data_Sheet_1.docx]

**Supplementary Materials**

**Etiological characteristics of Avian Influenza H11 Viruses Isolated from Live Poultry Market in Southeast Coastal Region in China**

Lina Jiang^1#^, Jiaming Li^2#^, Huan Cui^3#^, Cheng Zhang^3^, Yifei Jin^2^, Yingying Fu^2^, Ningning Ma^4^, Fei Tang^1^, Yidun Zhang^1^, Jing Zheng^1^, Li Li^1^, Bing Lu^2^, Zehui Chen^1*^, Zhendong Guo^3*^, Zhongyi Wang^2*^

1 Xiamen Center for Disease Control and Prevention, 681 Shengguang Road, Xiamen 361021, China

2 Beijing Institute of Biotechnology, 20 Dongdajie Road, Beijing 100071, China

3 Changchun Veterinary Research Institute, Chinese Academy of Agriculture Sciences, 573 Tulip RD, Changchun, 130117, China

4 Beijing Institute of Health Care, Dongan Road, Beijing 100071, China

#These authors contributed equally to this work

* Correspondence to: Zhongyi Wang (zhongyi_wang@foxmail.com);

Zhendong Guo (guozd@foxmail.com);

Zehui Chen (3846172@qq.com)

**Supplementary Table S1. PCR Primers used in this study**

| **Primer** | **Sequence (5’-3’)** |
| --- | --- |
| Uni12 | AGCAAAAGCAGG |


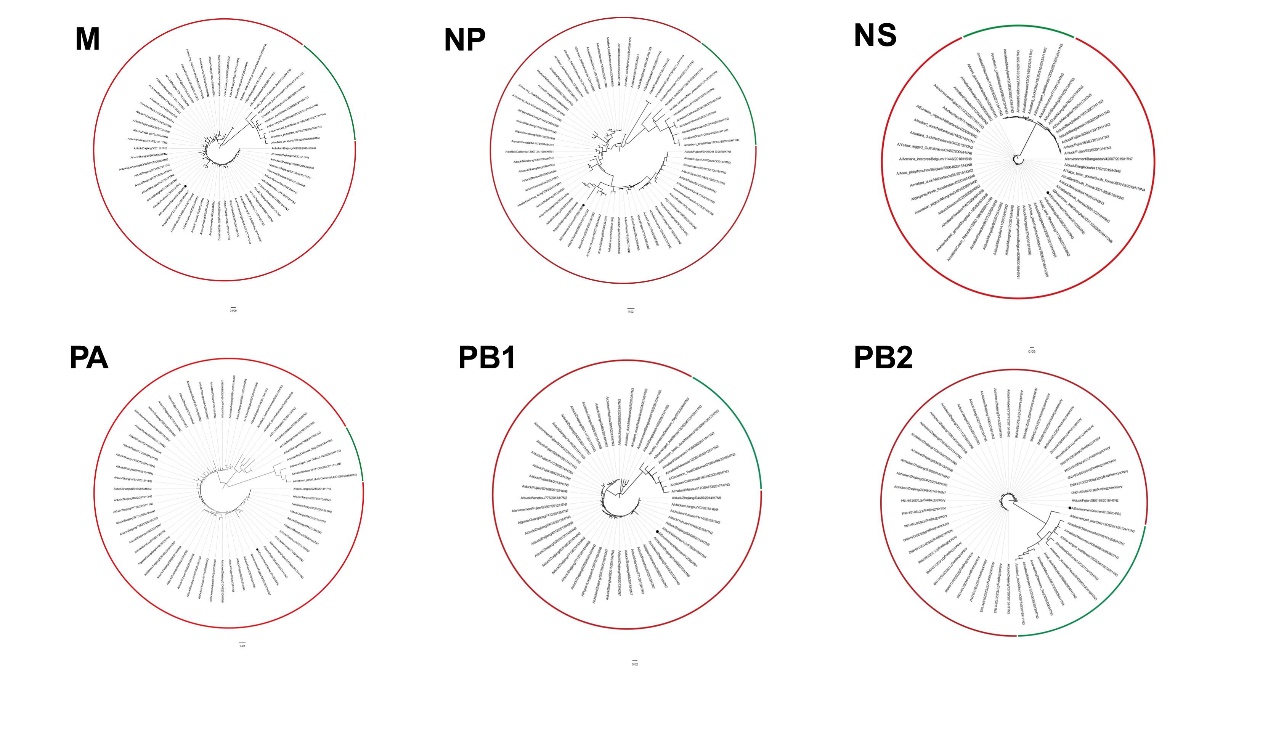


**Supplementary Figure S1 ML tree of M, NP, NS, PA, PB1 and PB2 of H11N3 viruses.** Phylogenetic trees were visualized using FigTree v1.4.4. Black dots indicate the isolate EV01 in this study.

**Supplementary Table S2. Receptor binding property of EV01, CA04 and HB777**

|  | **EV01** | | | |
| --- | --- | --- | --- | --- |
| **cRBCs** | | 2^7^ | 2^6^ | 2^5^ |
| **α2,3-cRBCs** | | 2^5^ | 2^4^ | 2^4^ |
| **α2,6-cRBCs** | | / | / | / |
| **Desial-cRBCs** | | / | / | / |

**Supplementary Table S3. Daily weight data for 14 days after infection**

|  | **Control (g)** | | | | | **EV01 (g)** | | | | |
| --- | --- | --- | --- | --- | --- | --- | --- | --- | --- | --- |
| **Day 0** | 16.9 | 17.3 | 18.5 | 17.5 | 16.2 | 17.6 | 18.3 | 16.5 | 17.2 | 18.1 |
| **Day 1** | 17.2 | 14.3 | 17.9 | 22.3 | 16.2 | 14.1 | 20.3 | 14.2 | 19.7 | 21.4 |
| **Day 2** | 21.1 | 19.2 | 21.4 | 12.5 | 16.7 | 13.2 | 21.6 | 16.2 | 20.1 | 19.6 |
| **Day 3** | 13.5 | 15.1 | 22.9 | 20.9 | 19.5 | 13.4 | 21.9 | 15.2 | 15.0 | 22.6 |
| **Day 4** | 19.7 | 16.0 | 16.1 | 20.3 | 21.2 | 12.2 | 19.4 | 18.9 | 19.2 | 15.6 |
| **Day 5** | 17.4 | 21.5 | 18.6 | 21.4 | 14.2 | 11.4 | 18.5 | 16.1 | 17.6 | 20.2 |
| **Day 6** | 21.1 | 15.8 | 23.3 | 20.8 | 14.1 | 20.4 | 21.8 | 16.3 | 12.5 | 14.2 |
| **Day 7** | 18.5 | 22.2 | 14.5 | 21.2 | 16.9 | 16.6 | 18.0 | 20.2 | 14.1 | 13.4 |
| **Day 8** | 19.8 | 22.2 | 14.5 | 21.2 | 16.9 | 15.4 | 16.5 | 21.7 | 14.4 | 15.5 |
| **Day 9** | 19.7 | 21.9 | 13.8 | 20.8 | 19.9 | 15.1 | 15.3 | 22.7 | 15.2 | 17.2 |
| **Day 10** | 14.4 | 23.1 | 22.4 | 22.5 | 15.2 | 22.4 | 16.9 | 14.9 | 14.4 | 20.6 |
| **Day 11** | 18.3 | 16.0 | 26.5 | 17.4 | 19.3 | 13.8 | 21.7 | 18.1 | 20.4 | 15.6 |
| **Day 12** | 15.7 | 24.2 | 19.2 | 19.3 | 17.7 | 17.6 | 17.9 | 13.8 | 17.0 | 24.7 |
| **Day 13** | 21.9 | 22.2 | 20.3 | 14.8 | 18.8 | 21.4 | 21.5 | 18.9 | 14.9 | 14.5 |
| **Day 14** | 21.7 | 24.2 | 22.6 | 15.4 | 16.3 | 23.4 | 15.1 | 15.0 | 19.4 | 19.7 |

**Supplementary Table S4. Virus titers in different tissues of mice at 1, 3, 5 and 7 dpi (Log10 EID_50_/mL)**

|  | **1 d.p.i** | | | **3 d.p.i** | | | **5 d.p.i** | | | **7 d.p.i** | | |
| --- | --- | --- | --- | --- | --- | --- | --- | --- | --- | --- | --- | --- |
| \| **Nasal Turbinate** \| \| --- \| | 1.95 | 1.20 | 0.95 | 1.20 | 1.20 | 1.20 | 1.45 | 1.95 | 1.20 | 1.45 | / | / |
| **Trachea** | 1.20 | 1.20 | / | 1.20 | 0.95 | / | / | / | / | / | / | / |
| **Lung** | 3.20 | 2.95 | 2.45 | 2.45 | 2.20 | 1.95 | 3.95 | 3.45 | 3.20 | 0.95 | 1.20 | 1.2 |
| **Liver** | / | / | / | / | / | / | / | / | / | / | / | / |
| **Brain** | / | / | / | / | / | / | / | / | / | / | / | / |
| **Heart** | 2.20 | 1.95 | 1.20 | 1.20 | 0.95 | / | 1.20 | / | / | / | / | / |
| **Spleen** | / | / | / | / | / | / | / | / | / | / | / | / |
| **Kidney** | / | / | / | / | / | / | / | / | / | / | / | / |
| **Pancreas** | / | / | / | / | / | / | / | / | / | / | / | / |
| **Intestine** | / | / | / | / | / | / | / | / | / | / | / | / |
